# Hospital outbreak caused by linezolid resistant *Enterococcus faecium* in Upper Austria

**DOI:** 10.1186/s13756-019-0598-z

**Published:** 2019-09-09

**Authors:** Heidrun Kerschner, Adriana Cabal, Rainer Hartl, Sigrid Machherndl-Spandl, Franz Allerberger, Werner Ruppitsch, Petra Apfalter

**Affiliations:** 1 National Reference Center for Antimicrobial Resistance and Nosocomial Infections, Institute for Hygiene, Microbiology and Tropical Medicine, Ordensklinikum Linz Elisabethinen, Fadingerstrasse 1, 4020 Linz, Austria; 2 AGES - Austrian Agency for Health and Food Safety, Institute of Medical Microbiology and Hygiene, Waehringerstrasse 25a, 1090 Vienna, Austria; 3 European Public Health Microbiology Training Programme (EUPHEM), European Centre for Disease Prevention and Control (ECDC), Stockholm, Sweden; 4 Department of Internal Medicine 1, Ordensklinikum Linz Elisabethinen, Fadingerstrasse 1, 4020 Linz, Austria

**Keywords:** *Enterococcus faecium*, Linezolid, Austria, Whole genome sequencing

## Abstract

**Background:**

*Enterococcus faecium* is part of the human gastrointestinal flora but may act as opportunistic pathogen. Environmental persistence, high colonization capability and diverse intrinsic and acquired resistance mechanisms make it especially successful in nosocomial high-risk settings. In March 2014, an outbreak of Linezolid resistant *Enterococcus faecium* (LR*Efm*) was observed at the hematooncology department of a tertiary care center in Upper Austria.

**Methods:**

We report on the outbreak investigation together with the whole genome sequencing (WGS)-based typing results including also non-outbreak LR*Efm* and susceptible isolates.

**Results:**

The 54 investigated isolates could be divided in six clusters based on cgMLST. Cluster one comprised LR*Efm* isolates of genotype ST117 and CT24, which was identified as the causative clone of the outbreak. In addition, the detection of four other clusters comprising isolates originating from hematooncology patients but also at other hospitals, pointed to LR*Efm* transmission between local healthcare facilities. LR*Efm* patients (*n* = 36) were typically at risk for acquisition of nosocomial pathogens because of immunosuppression, frequent hospitalization and antibiotic therapies. Seven of these 36 patients developed LR*Efm* infection but were successfully treated. After termination of the initial outbreak, sporadic cases occurred despite a bundle of applied outbreak control interventions.

**Conclusions:**

WGS proved to be an effective tool to differentiate several LR*Efm* clusters in an outbreak. Active screening for LR*Efm* is important in a high-risk setting such as hematooncology, where multiple introductions are possible and occur despite intensified infection control measures.

**Electronic supplementary material:**

The online version of this article (10.1186/s13756-019-0598-z) contains supplementary material, which is available to authorized users.

## Background

Enterococci are gram-positive bacteria found in the environment and as part of the human gastrointestinal flora [1]. They can act as opportunistic pathogens causing a broad range of diseases such as blood stream or wound-associated infections [2]. Hospital-adapted clones such as clonal complex (CC)17 *Enterococcus faecium* show persistence in the environment and high colonization capability [3]. *E. faecium* has diverse intrinsic resistance mechanisms to antibiotics and is able to progressively acquire antimicrobial resistances such as to ampicillin and vancomycin (VAN), thus limiting the therapeutic options [4]. One therapy of last resort against vancomycin-resistant Enterococci (VRE) is the oxazolidinone linezolid (LZD), which inhibits protein synthesis by binding to the 50S 23S rRNA [1]. Resistance to LZD has already been reported in *E. faecium* with the most common resistance mechanisms referring to mutations in the V domain of the 23S rRNA [5, 6]. Mutations in the sequence of genes encoding the riboproteins L3, L4 and L22 account for the second most common mechanisms. Third, recently described plasmid-mediated resistances due to *cfr* [7], *optrA* [8] and *poxtA* [9] and finally, yet unknown LZD resistance mechanisms are known to exist [10]. Risk factors associated with LZD resistance include transplants and surgery, immunosuppression and previous or ongoing treatment with LZD [11, 12]. Usually, LZD resistant *E. faecium* (LR*Efm*) strains emerge in patients 22–125 days after treatment [13]. LR*Efm* outbreaks tend to be mostly colonizations, although clinical outbreaks with invasive LR*Efm* infections have been reported [14].

## Methods

In March 2014, routine surveillance cultures of stool and urine from four patients hospitalized at the department of internal medicine 1 (DIM1) of an Austrian tertiary care center tested positive for LR*Efm*. An outbreak investigation was then initiated to identify the source of the outbreak, characterize the outbreak strain by whole genome sequencing (WGS) and antimicrobial susceptibility testing and apply control measures.

### Hospital and ward description

The DIM1 is one of three departments of internal medicine at a tertiary care center (hospital 4) in Linz, Upper Austria. It consists of a general oncology ward (16 rooms for 33 patients), a leukemia and autologous stem cell transplantation ward (8 rooms for 12 patients), a stem cell transplantation unit (5 rooms for 5 patients) and an outpatient clinic. The bed occupancy rate ranges between 90 and 100%.

### Outbreak description

Between March and May 2014, we identified ten LR*Efm* colonized patients and one patient with LR*Efm* bloodstream infection who were all hospitalized at the DIM1. The outbreak was contained in June 2014 through implementation of control measures. Active case finding detected twelve additional patients at the DIM1 and four at other units of hospital 4 till the end of the year 2017 (total number of cases: 27). Thirteen of these 16 patients were categorized as colonized, one patient treated at DIM1 had LR*Efm* bloodstream infection and two patients treated in other departments had a urinary tract infection and a surgical site infection, respectively. A case was defined as any patient with culture-confirmed LR*Efm* identified at hospital 4 from the beginning March 2014 onwards. Cases were included in this study until the end of 2017.

### Patients

In total, 36 patients were included in the study. Twenty-seven of those were the cases from hospital 4 as described above (24 were patients of the DIM1 and three were patients from other wards), who were designated as patient collective A. In order to gain insight into the occurrence of the outbreak strain in the local and Austrian hospital community, an additional nine patients belonging to six other hospitals (hospitals 1 to 3 and 5 to 7) in the provinces of Carinthia, Upper Austria and Vienna were included in the study and denominated patient collective B. Their isolates had been collected between 2012 and 2018. Demographic and epidemiological data such as patient outcome (death/survival), immune status, routine screening or clinical sampling, hospital contact in the previous year and other parameters were collected, anonymized and then analyzed using Microsoft Excel 2016 and SPSS statistical software version 22.0 (Chicago, Illinois). Data on the exposure to LZD in the 28 days prior to isolation of LR*Efm* and current antibiotic treatment (yes/no) was retrieved from patient records at the time of the first LR*Efm* isolation. If available, duration of LR*Efm* carriage was estimated from surveillance culture data. LZD consumption data of the DIM1 were extracted from the hospital pharmacy database and analyzed by AVS.webKess software [15]. We investigated at patient level (patient collectives A and B, *n* = 36) the acquisition of the outbreak strain and possible associations with their demographics and epidemiological characteristics using STATA 13 software.

### Isolates

Isolates (*n* = 54) were retrieved from cryobanks (Mast, Reinfeld, Germany) and cultured on blood agar (Oxoid, Wesel, Germany). Maldi-TOF analysis (Bruker Daltonics, Bremen, Germany) was used for species confirmation. LZD susceptibility testing was done using disk diffusion and, for the 45 LR*Efm* isolates, gradient testing (Biomérieux, Marcy L’Étoile, France) according to EUCAST criteria and breakpoints (resistant: > 4 mg/L or < 19 mm, respectively). Additionally, Tedizolid (TDZ) gradient testing was performed for all LR*Efm* (Liofilchem, Roseto degli Abruzzi, Italy) according to the manufacturer’s instructions. VAN susceptibility data were retrieved from the routine antibiogram. Finally, all LR*Efm* were investigated for the presence of *optr*A and *cfr* using previously published primer sets [16].

For comparison of WGS-based typing data, we included nine additional LZD susceptible *E. faecium* (LS*Efm*) isolates (eight from blood culture and one from stool) recovered from eight patients staying at hospitals 4 and 6 between May 2014 and June 2017 (patient collective C). Two of those were collected from patients having also an LR*Efm* isolate. The others were chosen because they were invasive isolates from affected wards.

### DNA extraction, WGS and typing

High-molecular-weight DNA from the 54 bacterial overnight cultures was isolated using a MagAttract HMW DNA kit (Qiagen, Hilden, Germany). DNA was quantified using DropSense 16 (Trinean NV/SA, Gentbrugge, Belgium). Library for WGS was prepared with a NexteraXT kit (Illumina, Inc., San Diego, CA, USA) according to manufacturer’s instructions and a 300-bp paired-end sequencing run was performed on an Illumina MiSeq instrument using the MiSeq V3 reagent kit (Illumina Inc., San Diego, CA, USA) for the 54 *E. faecium* isolates. Raw reads were de novo assembled into draft genomes using SPAdes version 3.11.1 [17]. *Contigs* were then filtered for a minimum coverage of 5 and minimum length of 200 base pairs. SeqSphere+ software (Ridom GmbH, Münster, Germany) was used for strain typing using a public core genome multilocus sequence typing (cgMLST) scheme [18]. Minimum spanning trees (MST) were generated to illustrate the number of allelic differences between isolates and visualize clusters. The allelic cluster threshold was set to ≤20 allelic differences as previously proposed [18]. Sequence types (STs) from the classical MLST [19] were in silico extracted from WGS data using SeqSphere+. Likewise, the three genes encoding the riboproteins L3, L4 and L22 and the 23S rRNA genes were extracted and screened for point mutations associated to LZD resistance by comparing their sequences with those of the *E. faecium* DO reference strain Antimicrobial resistance genes including those conferring LZD resistance (*optrA*, *poxtA* and *cfr* and its variants) were identified via the Comprehensive Antibiotic Resistance Database (CARD) [20]. In addition, both the point mutations and the genes conferring LZD resistance including G2576 U located in the 23S rRNA were double-checked using LRE finder [21]. The presence of specific virulence genes (VGs) for *E. faecium* among the isolates was investigated using the Virulence Factors Database and Virulence Finder 2.0 [22, 23]. Plasmid Finder was used for plasmid identification among the sequenced isolates [24]. Lastly, we assessed at an isolate level (*n* = 54) possible associations between the resistance phenotype (LR*Efm*/LS*Efm*) of the isolates and the presence of VGs, resistance genes and the point mutation 23S rRNA G2576U.

## Results

### Characteristics of all study patients and epidemiological data

**Fig. 1:**
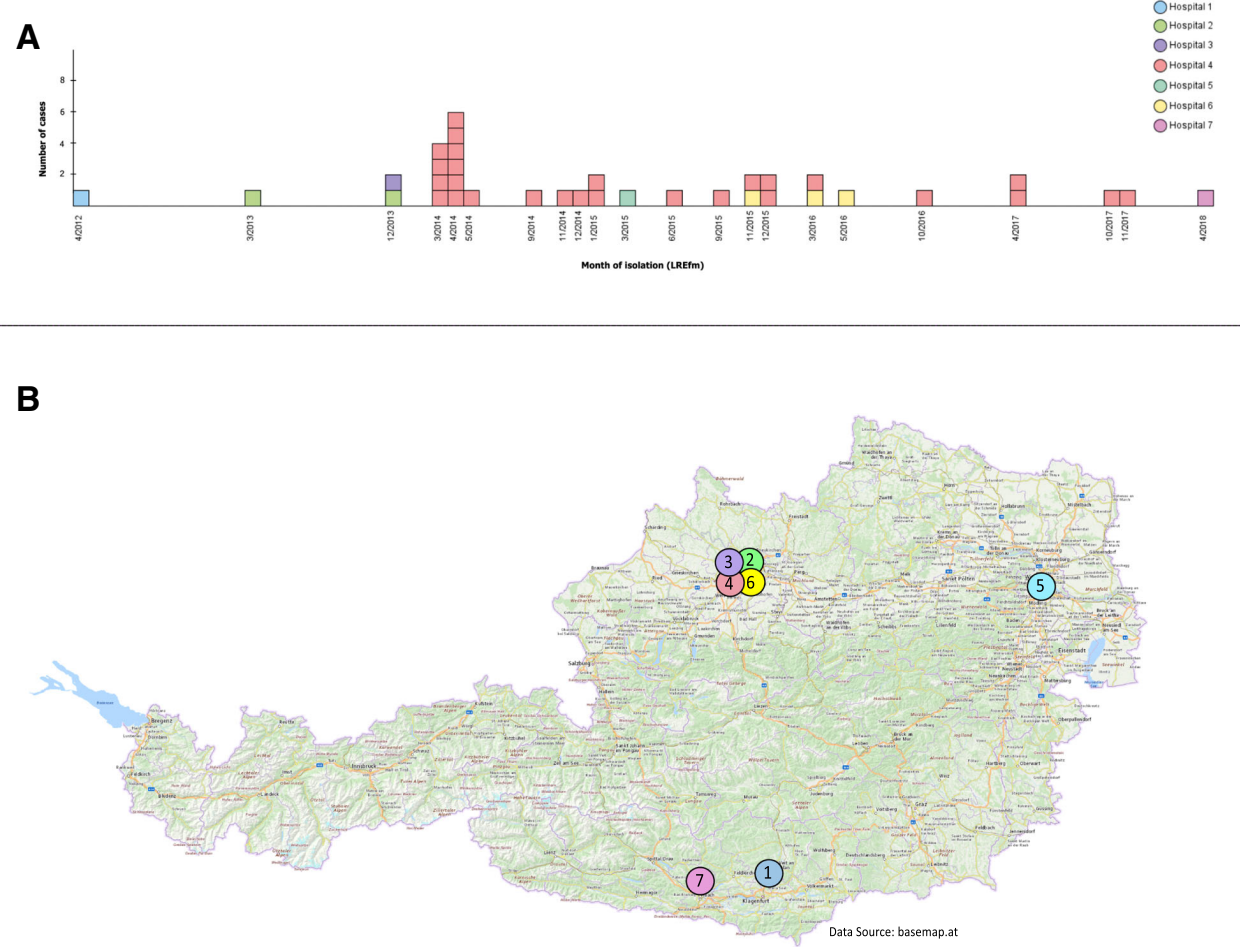
**a**. Epidemiological curve representing the 36 patients with a LZD resistant isolate within the period 2012–2018. **b**. Map of Austria with locations of the seven hospitals that provided LR*Efm* isolates

In the study period a total of LR*Efm* 36 patients were detected (patient collectives A and B). Their demographic and epidemiological characteristics are summarized in Table 1. Figure 1a shows the epidemiological curve of the outbreak by collection date of the first LR*Efm* isolate. Patients with more than one isolate are represented once only in the epicurve. The location of the hospitals is shown in Fig. 1b.

**Table 1:** Clinical and epidemiological data obtained for the 36 patients of collective A and B

Characteristics		
Age, years, median (range)	57 (22–91)	
Sex, n (%)		
Male	23	63.9
Female	13	36.1
Patient status, n (%)		
Inpatient	35	97.2
Outpatient	1	2.8
Transfer from other Hospital/LTCF, n (%)		
Yes	8	22.2
No	25	69.4
Unknown	3	8.3
LZD therapy (previous 28 days), n (%)		
Yes	10	27.8
No	25	69.4
Unknown	1	2.8
Duration of LZD therapy, days, median (range)	9 (3–35)	
LREfm status, n (%)		
Infection	4	11.1
Colonization	30	83.3
Unknown	2	5.6
Duration of colonization, days, median (range)	22 (3–245)	
Current antibiotic therapy, n (%)		
Yes	36	100.0
No	0	0.0
Hospital contact in previous year, n (%)		
Yes	31	86.1
No	2	5.6
Unknown	3	8.3
Underlying disease, n (%)		
Hematology-Oncology	25	69.4
allogeneic SCT	17	
autologous SCT	3	
Trauma/orthopedic	4	11.1
Abdominal surgery/Pancreatitis	4	11.1
Other	3	8.3
Immunocompromised, n (%)		
Yes	31	86.1
No	4	11.1
Unknown	1	2.8
Died during follow-up (until end of 2017), n (%)		
Yes	15	41.7
No	14	38.9
Unknown	7	19.4
Source material first LREfm isolate, n (%)		
Stool	18	50.0
Urine	9	25.0
Swab (wound, throat, eye)	7	19.4
Blood culture	1	2.8
Catheter tip	1	2.8
Source material secondary isolates, n (%)		
Stool	6	
Blood culture	2	
Urine	1	
Screening sample, n (%)		
Yes	24	66.6
No	11	30.5
Unknown	1	2.77

### Patient movement and clinical characteristics

**Fig. 2:**
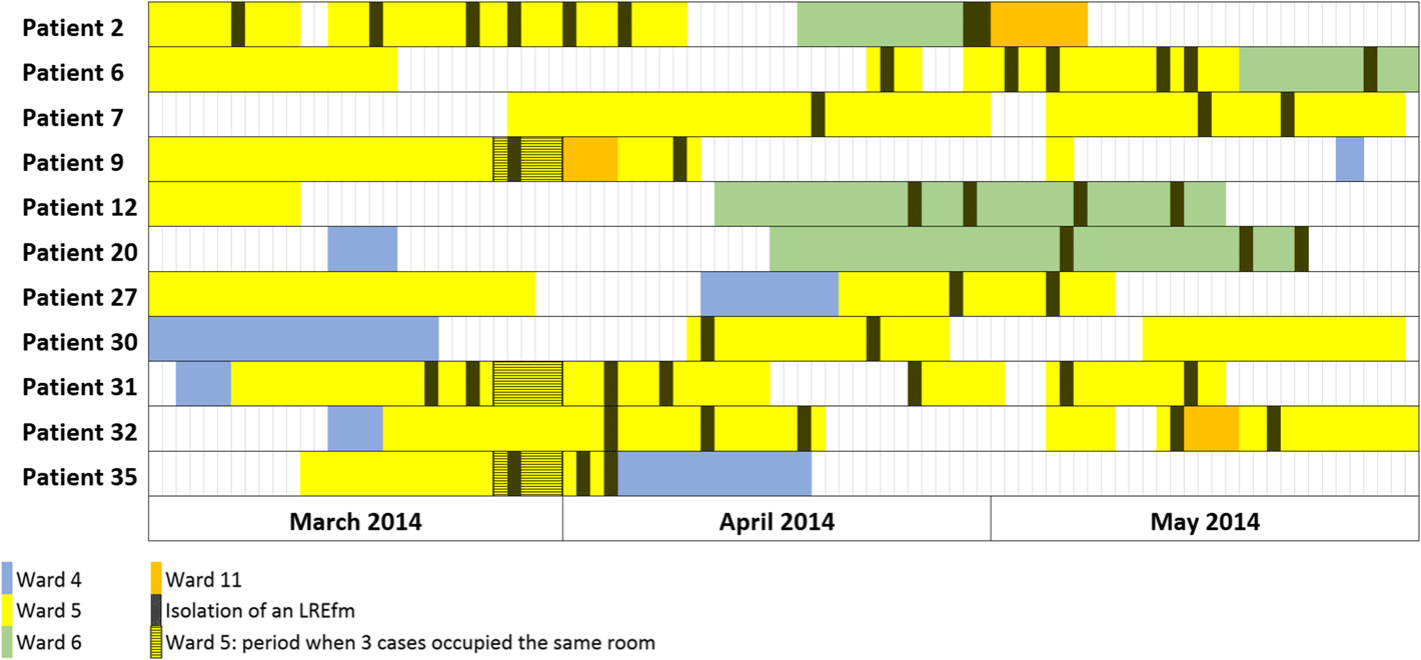
Patient trace at hospital 4 for the months March to June 2014. Each box corresponds to one day. The striped area marks the time when three of the first four cases occupied the same room on ward 5

When patient movement of cases (collective A) was traced, possible direct transmission could be shown for 10 out of 11 cases during the initial outbreak event (March 2014 to May 2014). Five of the eleven cases shared a room with confirmed LR*Efm* cases and another five were treated in wards at the same time as confirmed cases (Fig. 2).

Of the 16 additional cases detected between September 2014 and November 2017, six shared at least the DIM1 with known LR*Efm* carriers during their hospitalization. For ten cases, no direct epidemiological link could be established except for possible indirect transmission on previously affected wards.

Most patients (collective A + B) had multiple LR*Efm* isolates, typically from stool and urine, but eventually lost those enterococci again. To estimate duration of colonization, time from first LR*Efm* isolate until clearance was available for 21 patients and ranged from three to 245 days (median 22 days). For only two patients the same LR*Efm* clone could be cultured again after more than 1 year.

Five patients staying at hospital 4, one at hospital 6 and one at hospital 1 had an LR*Efm* infection (total *n* = 7), the rest was considered colonized. All seven infected patients were treated with VAN and none of them died. Fifteen (41.6%) patients died during follow-up but only two died during their stay at hospital 4 at the time of LR*Efm* detection of unrelated causes. All patients (100%) received antibiotics at the time of LR*Efm* detection. Ten (27.8%) patients had received LZD in the 28 days prior to the recovery of LR*Efm*, whereas 25 had not been exposed. Eight patients (22.2%), all from hospital 4, had been transferred from other hospitals, two of them from hospital 6. Thirty-one (81.1%) patients had been hospitalized in the previous year and the same number (81.1%) were immunocompromised. The majority of patients (*n* = 24, 66.6%) had been tested for LR*Efm* as part of screening.

### Antimicrobial susceptibility testing

The LR*Efm* isolates were all LZD resistant by disk diffusion testing and had a median MIC of 32 mg/L (range 4 to > 256). The two isolates with an MIC of 4 mg/L were included because of their LZD zone diameters of 17 and 18 mm, respectively. TZD MICs ranged from 1 to 32 mg/l (median 2 mg/L) and were all lower than the respective LZD MICs. Three isolates showed MICs of > 256 mg/l for VAN.

### WGS-based typing

**Fig. 3:**
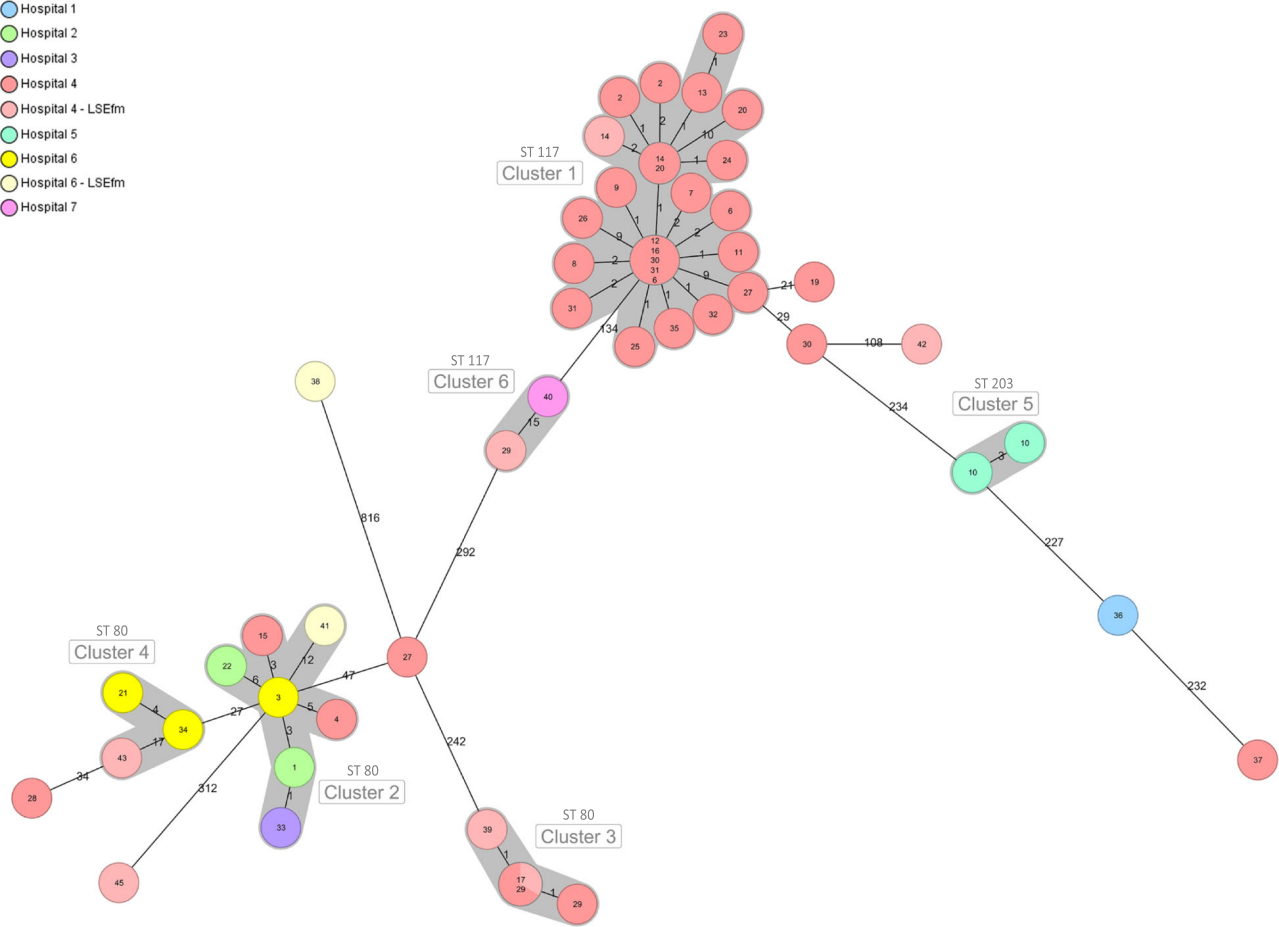
Minimum spanning tree showing the genetic relationship among the 54 sequenced isolates based on cgMLST. Each circle represents one or more isolates, color-coded by hospital of origin. Numbers inside each circle represent the patient ID. Shaded areas depict clusters. Connection lines indicate the number of allelic differences between the isolates. All isolates are LR*Efm* except those ones that are light-colored, which represent LS*Efm* isolates

WGS-based typing assigned the 54 isolates to seven sequence types (STs) (Table 2), with ST117 (*n* = 30; 55.5%) and ST80 (*n* = 18; 33.3%) being the most frequent. Within the LR*Efm* group (*n* = 45), ST117 was the predominant ST (*n* = 28, 62%) while for the LS*Efm* ST80 was the most frequent ST (*n* = 5, 56%). The vast majority of ST117 isolates belonged to cluster type (CT) 24 (*n* = 25, 83%). All the other STs were found once or twice. Based on the cgMLST data of the 54 isolates, the MST revealed six clusters (Fig. 3). We only identified one cluster (cluster 1) with 25 LR*Efm* isolates (46.3%, from 20 patients overall) as part of the outbreak at the DIM1 in hospital 4. An LS*Efm* isolate from a case also clustered in cluster 1. All isolates within the cluster were ST117 and all but one presented CT24. Isolates of cluster 1 differed by 0 to 10 alleles.

**Table 2:** Characteristics of the 54 *E. faecium* isolates based on patient data and molecular results

Case	Province	Hospital	Ward	Isolate	Isolation year	Source	Phenotype	LRE mechanism	ST	CT	Cluster number^a^	Linezolid MIC (mg/L)	Tedizolid MIC (mg/L)
1	UA	2	2	Ef-03	2013	Wound swab	LREfm	unknown	80	1873	2	32	4
2	UA	4	5	Ef-11	2014	Stool	LREfm	G2576U, A2598G	117	24	1	> 256	32
2	UA	4	6	Ef-22	2014	Blood culture	LREfm	unknown	117	24	1	32	4
3	UA	6	13	Ef-44	2016	Wound swab	LREfm	unknown	80	1873	2	64	2
4	UA	4	10	Ef-36	2015	Wound swab	LREfm	unknown	80	1873	2	128	4
6	UA	4	5	Ef-17	2014	Urine	LREfm	G2576U	117	24	1	32	8
6	UA	4	5	Ef-20	2014	Stool	LREfm	unknown	117	24	1	64	2
7	UA	4	5	Ef-15	2014	Urine	LREfm	unknown	117	24	1	16	4
8	UA	4	11	Ef-43	2016	Throat	LREfm	G2576U	117	24	1	32	2
9	UA	4	5	Ef-05	2014	Stool	LREfm	unknown	117	24	1	16	2
10	V	5	N/A	Ef-24	2015	Stool	LREfm	unknown	203	20	5	16	2
10	V	5	N/A	Ef-25	2015	Stool	LREfm	unknown	203	20	5	16	2
11	UA	4	5	Ef-40	2015	Stool	LREfm	unknown	117	24	1	32	2
12	UA	4	6	Ef-18	2014	Urine	LREfm	G2576U	117	24	1	32	2
13	UA	4	5	Ef-37	2015	Stool	LREfm	unknown	117	24	1	64	2
14	UA	4	6	13639	2015	Blood culture	LSEfm	unknown	117	24	1	n.d.	n.d.
14	UA	4	6	Ef-31	2015	Stool	LREfm	unknown	117	24	1	32	2
15	UA	4	9	Ef-30	2014	Urine	LREfm	unknown	80	1873	2	64	4
16	UA	4	5	Ef-28	2014	Urine	LREfm	G2576U	117	24	1	64	8
17	UA	4	6	Ef-35	2015	Stool	LREfm	unknown	80	16	3	64	4
19	UA	4	7	Ef-26	2017	Urine	LREfm	G2576U	117	1875	not clustered	64	8
20	UA	4	6	Ef-23	2014	Stool	LREfm	G2576U, A2598G	117	1872	1	32	4
20	UA	4	6	Ef-27	2014	Stool	LREfm	G2576U	117	24	1	32	16
21	UA	6	12	Ef-42	2016	Catheter	LREfm	G2576U, A2598G	80	315	4	64	4
22	UA	2	1	Ef-02	2013	Wound swab	LREfm	G2576U	80	1873	2	128	16
23	UA	4	5	Ef-39	2015	Stool	LREfm	unknown	117	24	1	4	1
24	UA	4	6	Ef-29	2014	Stool	LREfm	unknown	117	24	1	8	2
25	UA	4	5	Ef-48	2017	Stool	LREfm	unknown	117	24	1	16	1
26	UA	4	5	Ef-46	2017	Stool	LREfm	unknown	117	24	1	32	1
27	UA	4	5	Ef-19	2014	Urine	LREfm	unknown	117	24	1	32	2
27	UA	4	5	Ef-21	2014	Stool	LREfm	unknown	80	1879	not clustered	4	1
28	UA	4	5	Ef-47	2017	Stool	LREfm	unknown	80	1876	not clustered	32	2
29	UA	4	6	14921	2016	Blood culture	LSEfm	unknown	80	16	3	n.d.	n.d.
29	UA	4	5	Ef-32	2015	Stool	LREfm	unknown	80	16	3	64	2
29	UA	4	5	Ef-33	2015	Blood culture	LREfm	unknown	80	16	3	64	4
29	UA	4	5	Ef-41	2016	Stool	LSEfm	unknown	117	929	6	2	2
30	UA	4	5	Ef-13	2014	Stool	LREfm	unknown	117	1878	not clustered	16	2
30	UA	4	5	Ef-14	2014	Stool	LREfm	G2576U	117	24	1	8	2
30	UA	4	6	Ef-34	2015	Urine	LREfm	unknown	117	24	1	64	2
31	UA	4	5	Ef-07	2014	Urine	LREfm	unknown	117	24	1	16	2
31	UA	4	5	Ef-08	2014	Stool	LREfm	unknown	117	24	1	16	2
32	UA	4	5	Ef-12	2014	Stool	LREfm	unknown	117	24	1	16	2
33	UA	3	3	Ef-04	2013	Eye swab	LREfm	G2576U	80	1873	2	32	4
34	UA	6	16	Ef-38	2015	Blood culture	LREfm	unknown	80	315	4	32	2
35	UA	4	5	Ef-09	2014	Stool	LREfm	G2576U	117	24	1	16	2
36	C	1	1	Ef-01	2012	Wound swab	LREfm	unknown	192	1877	not clustered	16	2
37	UA	4	5	Ef-45	2016	Stool	LREfm	unknown	78	1874	not clustered	32	4
38	UA	6	15	14962	2016	Blood culture	LSEfm	n.a.	1479	not assigned	not clustered	n.d.	n.d.
39	UA	4	5	12981	2014	Blood culture	LSEfm	n.a.	80	16	3	n.d.	n.d.
40	C	7	14	Ef-51	2018	Urine	LREfm	G2576U	117	929	6	16	2
41	UA	6	15	14522	2015	Blood culture	LSEfm	n.a.	80	1873	2	n.d.	n.d.
42	UA	4	4	15549	2016	Blood culture	LSEfm	n.a.	1466	not assigned	not clustered	n.d.	n.d.
43	UA	4	7	15378	2016	Blood culture	LSEfm	n.a.	80	not assigned	4	n.d.	n.d.
45	UA	4	4	15925	2017	Blood culture	LSEfm	n.a.	80	467	not clustered	n.d.	n.d.

^a^Numbering allocated for each cluster in the MST

*n.a.* not applicable, *n.d.* not done

*C* Carinthia, *CT* Cluster type (cgMLST), *MST* minimum spanning tree, *ST* Sequence Type (classical MLST), *UA* Upper Austria, *V* Vienna

WGS-based typing revealed that 10 out of 11 cases (see also Fig. 2, all patients except patient 30 / Ef-13) detected at the beginning of the outbreak were part of it and therefore their isolates (*n* = 15) belonged to cluster 1, confirming the transmission suspected by patient movement data. After May 2014, nine cases were detected and these also carried the outbreak clone (*n* = 10 isolates, grouping in cluster 1). Last, seven additional cases from hospital 4 detected after May 2014 were not part of the outbreak, grouping five LR*Efm* isolates in clusters 2 (*n* = 2) and 3 (*n* = 3). At a patient level (*n* = 36), we found significant associations between acquisition of the LR*Efm* outbreak strain and screening sampling (Exact *p* < 0.005) as well as a fatal outcome during follow-up (Exact *p* < 0.005).

Clusters 2 to 6 comprised isolates from patients staying at different hospitals and from a wider period (2012–2018) (Fig. 3). Cluster 2 (ST80, CT1873) comprised two LR*Efm* isolates from two patients of hospital 4, not treated at the DIM1, four LR*Efm* isolates from four patients at hospitals 2 (*n* = 2), 3 (*n* = 1) and 6 (n = 1) and also one LS*Efm* isolate from hospital 6. None of these patients was treated for an oncological disease, they were typically older (median age 69 years) and two had been transferred from long term care facilities.

Cluster 3 (ST80, CT16) consisted of five isolates from four patients treated at the DIM1, of which two were LS*Efm*. Cluster 4 (ST80, CT315) grouped three isolates from three patients staying at hospitals 6 (n = 2) and 4 (n = 1), the latter being an LS*Efm* from a patient who had not been treated at the DIM1. Cluster 5 (ST203, CT20) grouped two isolates from a patient staying at hospital 5 in Vienna. Cluster 6 (ST117, CT929) comprised two isolates obtained at two hospitals located in different regions. One of them originated from hospital 7 in Carinthia and the other one was an LS*Efm* stool isolate from a DIM1 patient at hospital 4, from whom also three LR*Efm* isolates were included in the study all clustering in cluster 3. The timespan between obtainment of cluster 6 isolates was 2 years and there was no epidemiological data to explain their relatedness.

Last, a number of isolates did not cluster with any other isolate: five LR*Efm* from five patients at hospital 4, three LS*Efm* from two patients at hospital 4 and one at hospital 6 and one isolate from one patient at hospital 1.

Twenty-four resistance genes were detected among the 54 isolates (Additional file 1: Table S1). The most common ones were AAC (6′)-li (*n* = 52; 96.3%), *efm*A (n = 52; 96.3%) and *erm*B (*n* = 48; 88.9%). Moreover, the multidrug efflux pump *efm*A was detected among all LR*Efm* isolates within cluster 1. Significantly higher proportions were found for *dfr*F (Fisher’s exact *P* = 0.0020) and *sat*4 (Fisher’s exact *P* = 0.0499) within the LR*Efm* (*n* = 45).

PCRs targeting *cfr* and *optr*A genes were negative for all LR*Efm*. These genes, as well as *poxtA*, were also absent when blasting WGS data against the CARD database, the LRE tool from CGE server and Plasmid finder. Thirteen out of 36 (36.1%) cases presented at least one LR*Efm* isolate carrying the point mutation G2576U at the 23S rRNA, as revealed when using CARD database meaning that 25.9% (*n* = 14) of the *E. faecium* isolates carried that point mutation (Table 2 and Additional file 1: Table S1). The number of 23S rRNA mutated copies varied between 2 and 6. In addition, three of those isolates carried a novel point mutation at A2598G of the 23S rRNA. For 31 *LREfm* isolates, the resistance mechanism was not found.

The three VAN resistant isolates (the two ST203 isolates from cluster 5 and a non-clustered ST117 isolate) carried the gene *van*A.

Ten VGs were detected among the typed LREfm and LSEfm isolates (Additional file 1: Table S2). The most frequently found VGs were *sgr*A (*n* = 53, 98%), *acm* (*n* = 47, 87%) and *ecb*A (*n* = 33, 61.1%) and only *ecb*A was found to be significantly more frequent (Fisher’s exact *P* = 0.013) among LR*Efm* isolates. We did not detect associations between ST and any of the VGs tested.

### Outbreak control measures

**Fig. 4:**
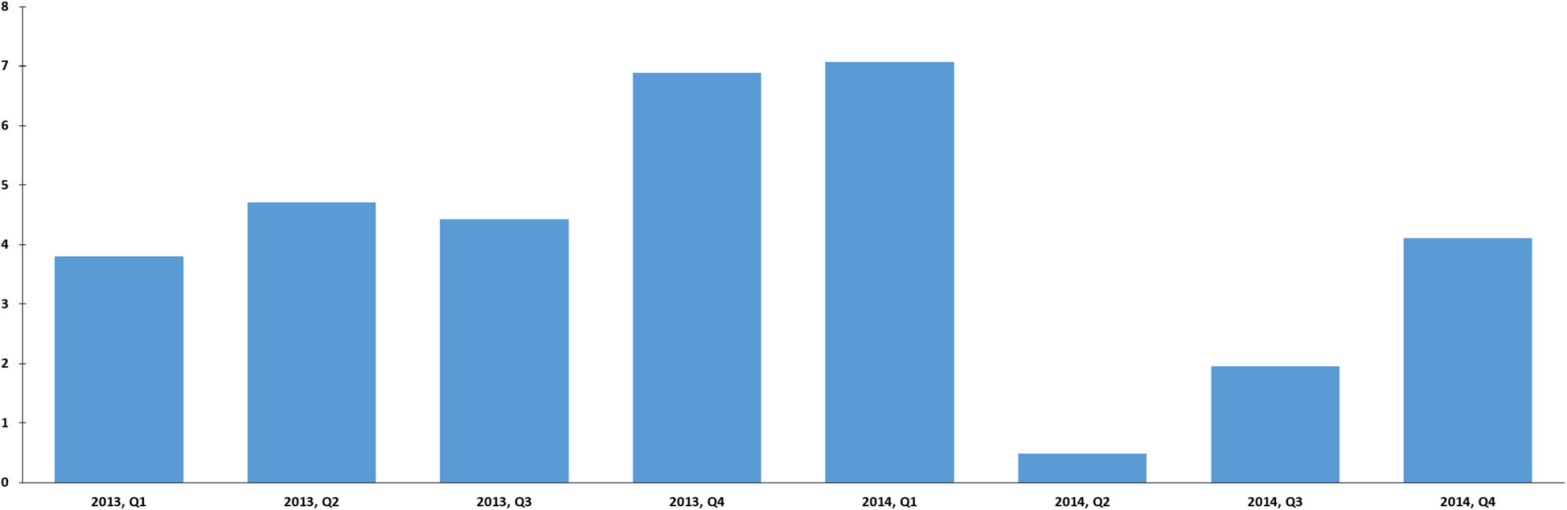
Linezolid use (p.o. and i.v.) in recommended daily doses (RDD) per 100 patient days at the DIM1 per quarter (Q) of the years 2013 and 2014

After the detection of the outbreak, active surveillance was put in place for the DIM1 including antimicrobial susceptibility tests for all clinical and screening *E. faecium* isolates. In addition, strict contact precautions for known colonized patients, single-room patient care and increased frequency of cleaning and disinfection of patient-near-surfaces especially in bathrooms was initiated. Moreover, LZD consumption was reduced by preferential use of VAN for gram-positive coverage in empiric antimicrobial therapy of neutropenic patients (Fig. 4**)**.

The outbreak was contained as of June 2014, however until the end of 2017 sixteen new cases were detected at hospital 4 at a rate of one per 2.6 months (Fig. 1). The fact that nearly half of these later cases were LR*Efm* clones other than the outbreak clone could be revealed by WGS only retrospectively.

## Discussion

In this study, we report the first LR*Efm* outbreak in an Austrian hospital, which involved colonized as well as infected patients. WGS identified a hospital-adapted ST117, CT24 LR*Efm* clone as the main causative agent of the outbreak in the affected hemato-oncology department. There was high clonality among this cluster, since all strains but one were ST117, CT24, and differed by ≤10 alleles. Interestingly, the outbreak clone did not spread to any other departments within hospital 4, meaning that the source was confined to DIM1. *E. faecium* ST117 is a widely disseminated clone that may present resistance to many drugs, including LZD [25]. In particular, ST117, CT24 has been previously identified in outbreaks caused by VAN-resistant *E. faecium* [26, 27] although up to our knowledge, this clone has been never associated to LR*Efm* outbreaks.

The addition of isolates from patient collectives B and C allowed us to gain more insight into the distribution of *E. faecium* between different healthcare facilities, especially for the region of Upper Austria. WGS also helped to elucidate that the outbreak at hospital 4 not just consisted of the cluster 1 clone (ST117, CT24), but also of two other clusters (2 and 3) and multiple unclustered isolates, suggesting several separate introductions of LR*Efm*.

Cluster 2 (ST80, CT1873) indicates a continuous spillover between four healthcare facilities located in the same province even though patient movement could not be analyzed comprehensively. Cluster 3 (ST80, CT16) represented a separated cluster within the DIM1, in which a patient showed loss of LR*Efm* during a one year period. Similarly, in cluster 4 (ST80, CT315) we could observe transmission of *E. faecium* between hospitals 4 and 6, although the isolate detected at hospital 6 was LS*Efm*, which might indicate the loss of the resistance trait in this strain. Concerning cluster 6 (ST117, CT929), available epidemiological data do not allow to understand why two isolates from different time periods and geographical areas clustered together.

The clustering together of LR*Efm* and LS*Efm* in nearly all the clusters is an interesting finding in our study and, to our knowledge, it has not been described previously. However, outbreak reports usually only include resistant isolates [28]. An explanation for our finding of genetically closely related LR*Efm* and LS*Efm* isolates may be the different antibiotic selective pressure in diverse hospitals.

All LR*Efm* cases received concurrent antibiotic treatment, which may account for selection of enterococci in the enteric microbiome. Recent exposure to LZD was found only in a minority (*n* = 10; 27.8%) of our cases, however, highlighting the contribution of environmental and person-to-person spread as previously described [25]. This is supported by the fact that for the majority of DIM1 cases close physical contact could be confirmed. Hemato-oncological patients generally are at high risk for infections, but with profound immunosuppression, frequent hospitalization and repeated administration of antibiotics they are especially prone to acquisition of multidrug-resistant *E. faecium* [29]. The association of acquisition of the outbreak strain with screening and death during follow-up, however, is most likely confounded by the intrinsic prognosis and type of care of this patient population.

All LR*Efm* isolates were clearly LZD resistant by disk diffusion testing. MIC testing yielded a wide range of concentrations but those were not associated with the number of mutated 23S rRNA genes, in contrast to previous reports [4, 5]. TZD MICs have been previously shown to be lower than LZD MICs in *E. feacium*, which was also the case in our study [30]. However, the clinical significance of this is unknown, since clinical breakpoints have not yet been defined.

Interestingly, a genetic basis for the phenotypic LZD resistance could not be identified in all isolates. The only resistance mechanism identified in 14 (31.1%) of the LR*Efm* in our study was the G2576U point mutation in the 23S rRNA, meaning that additional resistance mechanisms must exist among Austrian LR*Efm* isolates, as proposed elsewhere [31]. According to a recent systematic review [32], up to 80.5% of LR*Efm* carry the point mutation G2576U, although other authors have reported lower frequencies [10]. The previously undescribed additional point mutation A2598G is of unknown significance, however it was found only in three LR*Efm* and only in combination with the G2576U mutation. The plasmid-mediated LZD resistance genes *optrA, cfr* and *poxtA* could not be detected by PCR nor by WGS.

Regarding resistance mechanisms apart from ribosomal point mutations, in a recent study on *Mycobacterium abscessus* [33], authors identified efflux pumps *lmrS* and *mmpL9* at higher transcriptional levels among LZD-resistant isolates, suggesting an association with the resistant phenotype. Similarly, one could hypothesize that efflux pumps may contribute to LZD resistance, since *efm*A was detected among nearly all LR*Efm* isolates. In addition, *dfr*F, which encodes for a chromosomal dihydrofolate reductase [34], was significantly associated (Fisher’s exact = 0.002) with the LR*Efm* phenotype. Moreover, from a total of 11 cases that were treated with trimethoprim/sulfamethoxazole, eight cases belonged to the outbreak cluster. Trimethoprim is known to act against the reductase and sulfonamides have been described as a risk factor for LZD resistance acquisition [11].

Concerning the VGs, most of them (7/10) have been associated with more pathogenic *E. faecium* clones [25]. In accordance to other studies, *sgrA*, *acm, ecbA* are usually present at high frequencies in *E. faecium* strains of clinical origin [35, 36]. We did find an association between the adherence gene *ecb*A and the *LREfm* phenotype. Similarly, *ecb*A has been found in *E. faecium* outbreaks [37].

In our setting, the established control measures managed to terminate the initial outbreak event, although they did not prevent later sporadic cases. Unfortunately, environmental sampling was not performed and therefore an environmental reservoir was not identified. Nevertheless, the persistence of enterococci, especially of ST117, in hospital settings is well known [38] and as it has been shown here, high-risk populations such as hemato-oncological patients account for most of the cases. Fortunately, most patients were only temporarily colonized with LR*Efm* and only a minority of them developed infection.

A limitation of our study was the lack of systematic testing for clearance of LR*Efm* carriage, although our data suggest that in most patients LR*Efm* do not seem to become established for a longer time period. Nevertheless, it is important to promptly detect even LR*Efm* colonization since such patients may be a reservoir of transmission to patients of the same ward, the same hospital or even within a healthcare network. Also, colonization generally precedes invasive infections which may be difficult to treat, especially when other resistance mechanisms are present.

## Conclusions

We have described an outbreak caused by Linezolid resistant *Enterococcus faecium (*LR*Efm*) in an Austrian hematooncology unit. Whole-genome sequencing was a useful method to investigate isolates from the outbreak hospital and to compare them with strains from other healthcare facilities in Austria. The outbreak-causing strain was identified as highly clonal hospital adapted ST117, CT24 LR*Efm*. Multiple additional clusters were identified, partly explaining the perceived repeated occurrence of LR*Efm* after termination of the outbreak by multi-modal infection control measures. In a high-risk setting, active surveillance of LR*Efm* is important for prompt outbreak detection.

## Additional file

Additional file 1: **Table S1.** Resistance genes and point mutations at the 23S rRNA found among the sequenced strains. **Table S2.** Virulence genes carried by the 54 sequenced strains and resistance phenotype. (XLSX 17 kb)

## Data Availability

The raw WGS reads were deposited into Sequence Read Archive (SRA) database under NCBI accession PRJNA541232. The datasets used and/or analyzed during the current study are either included in the published article and its supplementary files or available from the corresponding author on reasonable request.

## Abbreviations

CC: clonal complex
cgMLST: Core genome multi locus sequence typing
CT: cluster type
DIM1: Department of Internal Medicine 1
EUCAST: European Committee on Antimicrobial Susceptibility Testing
LR*Efm*: Linezolid resistant *Enterococcus faecium*
LS*Efm*: Linezolid susceptible *Enterococcus faecium*
LZD: Linezolid
ST: Sequence type
VAN: Vancomycin
VRE: Vancomycin resistant enterococci
WGS: Whole genome sequencing
